# Family Relationships and Depression among Elderly Korean Immigrants

**DOI:** 10.5402/2011/429249

**Published:** 2011-06-16

**Authors:** Young-Me Lee, Karyn Holm

**Affiliations:** Nursing Department, DePaul University, 990 W. Fullerton Avenue, Suite 3000, Chicago, IL 60614, USA

## Abstract

The purposes of the study were to describe family relationships within the context of living arrangements (living with adult children or without adult children) and support network, and to further determine associations of these factors to depression in elderly Korean immigrants. Over 70% (*N* = 160) of Korean elders were found to live apart from their adult children. However, Korean elders who were living independently reported higher levels of depression in spite of their expressed desire to live independently and to be less dependent upon their adult children. These findings suggest that family support and close relationships with their adult children play a central role in adjusting to a new life and in preventing and/or lessening depression in elderly Korean immigrants.

## 1. Introduction

Koreans are one of the most rapidly growing immigrant groups in the United States. The passage of the Immigration Reform Act of 1965, which gives priority to family reunification among immigrants [1–3], contributes to why Koreans have been attracted to the United States at a rate faster than all other national groups with the exception of Mexicans and Filipinos. According to the 2008 U.S. Census Bureau American Community survey, there were over 1.5 million Koreans of all ages living in the United States, making them the fifth largest Asian-American population after Chinese, Filipino, Vietnamese, and Asian Indian.

The Immigration Reform Act of 1965 allowed immigrants who obtained citizenship after five years of residency to invite their elderly parents to apply for permanent residence. Therefore, the majority of Korean elders have immigrated into the United States at the invitation of their adult children [4]. They decided to migrate to the new country to live with their adult children even though they knew that immigration in later life was not an easy process. The family reunion features of immigration policies along with the traditional Korean family values serve to steadily accelerate the growth of the elderly Korean population in the United States. 

In the Korean culture, the family is the center of all social organizations. The family is considered a natural and spontaneous support system. It is characterized by natural attachment and reciprocal obligation, which serve various functions of support to all family members. At the core of the Korean family system is respect for elders and filial piety. Elders are valued for their experience and wisdom; they are consulted for advice, support, and resolution of family conflicts. This practice is strongly influenced by the Confucian tradition of filial piety [5]. It emphasizes that adult children care for their elders at home is in accordance with filial devotion and obedience. Respect for elders and support of the aged are social values that promote caring relationships between adult children and older parents. In this cultural practice, adult children have a strong responsibility to support and invite their parents to live with them once their parents become old and weak [5]. 

Previous studies have reported that ethnic elders, including Chinese, Japanese, and Koreans, face challenges as they try to maintain their traditional family values within the American cultural context. Ishii-Kuntz [6] and Kamo and Zhou [7] suggest that this traditional family value continues even when people migrate to a new society where family relationships and living arrangements are different. They found that family members were very involved in support of their aged parents among Asian immigrant groups that have a traditional history of strong family relationships and thus, concluded that ethnic elders preferred to live with their children, and that this is a cultural preference and expectation. Koh and Bell [8] also found in their study of Korean elders in the United States that the preferred sources of help in dealing with problems among elderly Korean immigrants were their spouses and children. This research suggests that living arrangements and family support of elderly immigrants in the United States are still greatly influenced by filial responsibility [6–8].

Unlike the studies discussed above, other research reported changes in family values of coresidence and family support. Lam et al. [9] revealed that elderly Chinese immigrants who migrated to the United States received less family support from their adult children than they had expected. This was a difficult emotional issue for the immigrant elders who still cherished family solidarity. In addition, those elderly immigrants who relied heavily on their adult children experienced more stress than those who were not dependent [10]. Another pilot study conducted by Lee [11] reported that the majority of elderly Korean immigrants felt a strong sense of shame, failure, powerlessness, and embarrassment because of their heavy dependence on their adult children. They also expressed concerns about having a poor relationship with their adult children. These conditions may cause elderly immigrants to live independently without seeking family support from their children. The elderly immigrants also perceived this as a major life stressor while living in the United States. Previous studies suggest that the stress of changing family relationships experienced by elderly Korean immigrants may contribute to increased psychological distress such as depression. 

Depression in later life is more serious because it is related to increased mortality from both suicide and medical illnesses [1, 4, 12, 13]. Research suggests that the risk of death from suicide is two times greater in the elderly population than in the general population. Specifically, elderly Korean immigrants in the United States have been reported to have a higher level of depression than other elderly Asian immigrants. Studies have shown the prevalence of depression among elderly Korean immigrants to be about twice that of the rate among the Chinese, Filipinos, and Japanese groups [1, 4].

Unfortunately, the literature contains conflicting perspectives, and the majority of studies examining family support networks in minorities were done nearly 20 years ago. However, these previous studies suggest that separate living arrangements and decreasing family support for elderly Korean immigrants will be an increasing trend. Despite this, there is a paucity of empirical data supporting the impact of such changes. More empirical data is needed to gain a comprehensive understanding of family relationships experienced by this group.

The purposes of this study were to describe family relationships associated with living arrangements and family support networks and to determine the associations of these factors to depression in elderly Korean immigrants.

The following are the research questions for this study.

- What are living arrangements and support networks in the U.S reported by elderly Korean immigrants?
- What are the differences in living arrangements and support networks between elderly Korean immigrants who live with adult children or without adult children?
- What is the association between family relationships (living arrangements and support networks) and depression among elderly Korean immigrants?

The theory of stress and coping [14] and acculturation theory [15] guided this research. According to Lazarus and Folkman [14], stress is viewed as a condition or feeling experienced when individuals perceive that they cannot adequately deal with the demands being placed on them or with threats to their well-being. Stressors refer to the environmental events or changes that give rise to stress [14, 16, 17]. 

Acculturation theory refers to the changes that groups and individuals undergo when they come into contact with another culture [15]. According to this theory, immigrants may experience tremendous demands while adjusting to the new culture. Such demands engender a unique type of stress referred to as *acculturative stress* [15]. Stress resulting from cultural adjustment may cause psychological discomfort and contribute to the development of various mental health problems, such as depression, among immigrants [4, 15, 18]. 

Studies have shown that stress alone cannot cause stress-linked diseases like depression. Other conditions such as inadequate cognitive appraisal or inadequate social resources additionally have to be present in order for the stress to lead to an illness [14]. Social resources can act to influence the person's coping behaviors. Social resources represent the individuals' social network and social support, which can be mobilized to deal with a stressful situation and may mediate stress-related outcomes [14]. 

Social support refers to the various types of aid or assistance that people receive from others. A key element of social support is how an individual undergoing stress perceives others as supportive or unsupportive [14, 19]. The support network refers to structural properties of social relationships and is typically measured by the number of people around the individual [14, 19]. Because there are limited studies exploring family relationships as perceived by elderly Korean immigrants, it was considered the stressor. Family relationships in terms of living arrangements and support networks were examined to understand the relationship between the stress-related phenomena and their impact on depression among this population, as proposed by stress and coping theory [14].

## 2. Methods

### 2.1. Research Design

This study utilized a descriptive comparative research design to explore family relationships associated with living arrangements and support network, in addition to the relationships between these family factors and depression. Comparisons were made between those elderly living with adult children and those not.

### 2.2. Sample and Setting

The sample consisted of 160 elderly Korean immigrants. Selection criteria were; (1) 65 years of age or older; (2) able to speak, read, and understand Korean; (3) no obvious cognitive impairments; and (4) noninstitutionalized. In order to obtain an adequate sample size, the Korean Senior Center was selected as the major sampling site. This senior center provides various services to Korean elders in a large Metropolitan community, including in-home services, individual counseling, public policy advocacy, language and other training services, cultural arts activities, and outreach programs.

### 2.3. Subject Collection Procedure

Before any data was collected, approval for the study was obtained from the Institutional Review Board at Rush-Presbyterian-St. Luke's Medical Center and from key administrators at the Korean American Senior Center. The subjects were recruited from June to August 2002 at the Korean American Senior Center. As this study did not involve any treatments or drug tests that may cause harm and was designed to utilize an anonymous survey, the Institutional Review Board determined that this study did not require a signed consent from the participants. After receiving verbal informal consent from the Korean elders, individual face-to-face interviews in Korean lasting approximately for one hour were completed.

### 2.4. Measures

Family relationship was measured by examining two major components: the elderly Korean immigrants' support networks and living arrangements. Support networks were measured by using the Personal Resources Questionnaire (PRQ), Part I [19] to assess family support. This instrument contains 12 life situations with which an individual may need help, such as financial aid. It also asks participants to identify available support resources, for example, spouse, adult children, and their satisfaction with the assistance that was provided by those individuals. Test-retest reliability of Part I yielded a score of 0.81 over a 4- to 6-week period for a sample of 100 adults [19]. A Cronbach's alpha coefficient was 0.78 in the present study.

In addition to support networks, ten questions related to living arrangements were included to explore family relationships. These questions were developed to elicit information about current living arrangements (living with children or without children), preferred living arrangements (living with children or without children), the reasons for the preference, and frequency of interaction with adult children. These questions were piloted with six individuals, representing the target population. 

Depression was measured using the translated Center for Epidemiological Study-Depression Scale ((CES-D-K) Radloff [20]). The CES-D-K is a self-report 20-item scale. The response format for each item is a four-point Likert-type scale ranging from zero to three. The possible overall score ranges from 0 to 60, with a cutoff of 16. Its scores indicate the level of depression that is diagnostically associated with CES-D score: 15 or less indicate no depression; 16–23 mild depression; 24 or higher clinical depression [20, 21]. A score of 16 or greater is indicative of clinically significant depressive disease. The Cronbach's alpha coefficients include 0.89 for 262 middle-aged Korean immigrant women [22], 0.87 for 200 older Korean immigrants [13] and 0.80 for the 160 elderly Korean immigrants in the present study.

### 2.5. Translation Procedure

All instruments had been previously translated to Korean from English. The instruments were tested with two Korean elders who read the Korean versions of all the instruments to ensure that they were easily understood and culturally sensitive.

### 2.6. Data Analysis

The data was collected, coded, and entered into a computer data file using SPSS software. Descriptive statistics, *t*-tests, and analysis of variances (ANOVAs) were calculated to analyze the demographic information and the variables related to family relationships.

## 3. Results

### 3.1. Sample

A total of 160 elderly Korean immigrants including 48 men (30%) and 112 women (70%) participated in this study. Age ranged from 69 to 91 years with a mean age of 74 years (SD = 6.7). Over 70% had arrived in the United States when they were 50 years or older. Thirty-four percent (*N* = 55) reported a gross yearly income of $0 to $4,999 per year and 55% reported $5,000 to $14,999. Over 50% (*N* = 80) reported that supplemental security income (SSI) was their major source of income, whereas only 2% reported the Social Security Administration (SSA) as their major source of income. Over 70% (*N* = 124) identified themselves as Protestant. A majority of the sample perceived their health as fair (31%) or good (38%). Forty-two percent (*N* = 68) indicated that elementary school was the highest completed level of education or that they had no formal education. Thirty-eight percent (*N* = 61) had graduated from high school in Korea.

### 3.2. Description of Family Relationships among the Sample

#### 3.2.1. Living Arrangements

Twenty-eight percent (*N* = 45) lived with their adult children, and the remaining 72% (*N* = 114) lived separately from their adult children (Table 1). This is much higher than Mui's [4] findings that 49% of that elderly Korean sample lived separately. However, this is lower than the Administration of Aging [23] report of the general elderly population (90%) living independently. 

#### 3.2.2. Housing

More than half of the respondents (54.4%) lived in senior apartment buildings. Twenty-five percent of the respondents lived in a private house or apartment. The last group (19.4%) lived in a house or apartment with their adult children.

#### 3.2.3. Satisfaction with Current Living Arrangements

As shown Table 1, more than 90% of the respondents were satisfied with their current living arrangements. Only a few respondents (*N* = 12) answered that they were dissatisfied with their current living arrangements.

#### 3.2.4. Different Living Arrangement If in Korea

Korean elders were asked whether their living arrangements would be different if they were in Korea. Forty-four percent of the respondents reported that the living arrangements would be different, whereas 56% said there would be no difference even if they were in Korea. 

#### 3.2.5. Preferred Living Arrangements

The respondents were also asked about preferred living arrangements, with or without their adult children. In this question, 77.5% answered that they would prefer to live alone, while 22.5% of the respondents would prefer to live with their adult children.

#### 3.2.6. Reasons for Living in Separate Households

The Korean elders who expressed a desire to live in a separate household were asked about their reasons for wanting to live separately. Forty respondents (25%) wanted to live independently to protect their privacy and freedom, 20 respondents (12.5%) thought it was less of a burden on their adult children, and 36 respondents (22.5%) answered that both were true for them (Table 1).

#### 3.2.7. Reasons for Living with Their Adult Children

Thirty-six respondents expressed a desire to live with their adult children, and they were then asked why they preferred this. Nine of the respondents answered because of the opportunity to be with their grandchildren. Six respondents said that adult children still needed their help, and five answered they want to live with their adult children because of loneliness. Furthermore, four respondents said that they preferred living with their adult children because of their children's filial responsibility to care for parents, and four others answered that they needed the support of their adult children.

#### 3.2.8. Support Networks

Data related to support networks was collected using the Personal Resource Questionnaire (PRQ85), Part I. It describes 12 situations to identify available support resources. Korean elders were asked to choose available support resources in response to specific situations.  Table 2 presents the most frequently named people in the social support networks identified by these Korean elders. Adult children, spouses, and friends were the most common members in their support networks. The majority of the respondents also identified that they had “no one” (no one available or preferred to handle it alone) to assist them in many situations that required support. 

In addition, the respondents reported that adult children (47.2%) were the most common source of support when they needed instrumental support for things such as financial problems, transportation, and emergency situations. However, in the case of emotional support, others (22.3%) and spouses (16%) were the most common support resources among Korean elders. Around 40% of the respondents identified that they had no one available to provide emotional support.

#### 3.2.9. Support Networks between Two Groups (Living with Children or without Children)

The differences in social networks as identified by the two groups living with or without children were analyzed. Both groups of respondents sought out their adult children first when they needed instrumental support. The results here showed a distinctive structure with the highest reference to the adult children and relatively less reference to other relationships. However, sources of emotional support for these Korean elders were more diversely structured than those of instrumental support. When they faced emotional problems such as being upset or lonely, both groups sought either non-family ties, friends, or solved the problems by themselves instead of seeking help from their adult children.

Even though the two groups shared some similarities in seeking instrumental and emotional support, some differences were identified. Respondents who lived with their adult children considered their adult children as a major source of support. For example, they sought help from their adult children when they had situations that required support such as buying medication (71.7%; 23.7%) and transportation problems (71.7; 32.5). Networks providing support for this group were structured primarily with adult children, and this may reflect that family support remains fundamental and essential for Korean elders living with adult children. However, when this group had emotional problems such as loneliness or other personal concerns, they usually kept that to themselves more than the group living independently. The group living independently had various people in their support networks including friends and neighbors for help with their emotional problems. Therefore, this finding suggests that Korean elders living independently might be more inclined to accept such help from sources other than family members, varying in degree according to the form of help sought. 

#### 3.2.10. Depression

The CES-D-K depression scale revealed that the mean CES-D-K score for this sample of 160 respondents was 12.21 (SD = 8.3) higher than that of Radloff's [20] general sample (9.20), higher than that of Noh and Avison's [18] study of the Korean sample in Canada (10.6), higher than that of Gupta and Yick's [24] study of older Chinese immigrants (11.7), and higher than Callahan et al. [25] study of the general elderly population (7). The mean score of this sample was a little slightly below the cut-off score of 16, which represents mild depression. However, 22.6% (*N* = 36) of this sample had scores of 16 or greater, indicative of clinical depression (Table 3). This is higher for the elderly Korean immigrants compared with the general elderly population (13%) and older Chinese immigrants (14%) [24, 25]. 

#### 3.2.11. Differences between the Family Relationships and Depression

*T*-tests and ANOVAs were used to compare differences in depression by variables related to family relationships (living arrangements and support networks). Korean elders who lived with their adult children showed lower CES-D scores than the group who lived independently (*t* = 2.669, df = 126, *P* = .009). In addition, this analysis also found that Korean elders, who identified their adult children as major support networks in regarding to feeling lonely, showed higher levels of CES-D scores than their counterparts. This finding may indicate that Korean elders received less assistance from adult children with their emotional problems.

## 4. Discussion

Many of the elderly Korean immigrants who participated in this study had arrived in the United States at an older age (age 50 or older). This may have made it more challenging to master daily living skills such as learning a new language, earning money, and adapting to the new culture. This sample had much lower income levels than the general elderly population. In addition, over half of the participants were totally reliant on supplemental security income (SSI) from the United States government, which was/is much higher than 38% of the general elderly population who receive SSI benefits [26]. This suggests that the elderly Korean immigrants in this study may not have had enough time or an opportunity to earn money to support themselves in the United States. Low income may prevent elderly Korean immigrants from living independently, leading them to rely heavily on their adult children for support after immigrating to the United States.

The elderly Korean immigrants in this study were found to have higher total depression scores than the general elderly population and the elderly Chinese immigrants as measured by the CES-D [24, 25]. More seriously, the percentage of the elderly Korean immigrants with depression scores of 16 or greater, indicating clinical depression, was greater than the general elderly population and the elderly Chinese immigrants. Consistent with other studies, this sample reported higher depressive symptoms for elderly Korean immigrants than for similar populations of general elderly or Korean nonelderly women [2, 5, 27]. 

As discussed earlier, elderly parents living with their adult children is an important cultural value in the tradition of the Korean family. However, over 70% of the participants in this study revealed that they lived apart from their adult children. This is lower than the Administration of Aging's [23] report which states that 90% of the general elderly population lives separately or independently of their children. Conversely, it is much higher than Mui's [4] findings indicating that 49% of their elderly Korean sample lived independently of their children. In addition, the majority of elderly Korean immigrants in this present study expressed a desire to live independently and not be a burden to their children. This finding was consistent with a previous investigation, which reported that over 70% of the Korean elders in a New York City sample wanted to live independently [8]. These findings may indicate that elderly Korean immigrants experience changes and accept these changes in a cultural pattern of living with and depending upon their adult children unlike previous studies. 

In spite of these changes in living arrangements, the adult children were still the main sources of support, providing both instrumental and emotional support for their elderly parents. This study supports previous conclusions that family and family networks are essential for Korean elders as documented in an earlier study [2]. This higher level of family support may be due to those Korean adult children who hold on to some of their own cultural tradition regarding family support for their elderly parents. It may be that adult children may be the only support networks that elderly Korean immigrants rely on in the United States. 

Another surprising finding was that Korean elders living independently had larger support networks consisting of friends or neighbors than the group of Korean elders living with their children. After Korean elders moved out from their adult children's homes, they learned how to live in their new environment without their adult children. These findings seemed to reflect the movement among Korean elders into the expansion of their support networks beyond the boundary of the family support network. Such a movement would then indicate the efforts of the elderly to cope with adaptation demanded of them in the process of dynamic social changes within and around the family network. 

These present findings reveal that living arrangements have a strong relationship with the level of depression among elderly Korean immigrants. Korean elders who lived with their adult children exhibited less depression even though a large number of them preferred to live independently. Being with adult children seemed to provide Korean elders with security, worthiness, and a sense of identity. This finding suggests that family is a critical and powerful factor that affects the emotional well-being of elderly Korean immigrants. An expressed desire for separate households and less dependence upon their adult children may be viewed as a way of decreasing cultural conflicts with their adult children and accepting a change in traditional family values. 

Surprisingly, Korean immigrants who were actually living independently seemed to have a higher level of stress, depression, and difficulty handling daily living even though they perceived themselves as having larger support networks. Korean elders living separately may develop numerous but not necessarily close relationships with others. Poor social integration and superficial relationships in this group may actually increase their level of depression. In addition, the readjustment process after moving away from their adult children, including mastering daily living skills, such as paying bills, might make them experience more stress and depression than the group living with their adult children. It seems that living with children provides Korean elders with higher quality relationships even though they received less support than they expected. Therefore, the findings from this analysis suggest that the size of the support networks may not be as important as the closeness and cohesiveness of family networks, as critical and powerful factors that affect mental well-being among elderly Korean immigrants.

Limitations of this study include use of a convenience sample, single-informant self-report methodology, and a cross-sectional design. In addition, the subjects in this study were recruited from the Chicago area, so these findings should not be generalized to other Korean groups living in different areas. In addition, most of the respondents participating in this study were living separately from their adult children. Thus, future study should include more Korean elders living with their adult children to better understand their perceptions of family relationships. 

### 4.1. Implications for Practice

This present study aids in understanding the trend of separate living arrangements and changes in family support for this population. Living independently for Korean elders seemed to be a very stressful experience regardless of the fact that they had more diverse structure for help than their counterparts. As elderly Korean immigrants are a rapidly growing population in the United States, we may see more Korean elders who live independently, which may also mean an increased prevalence of depression among this population. As health care providers, we need to be aware of an evolving trend in separate living arrangements and increase our awareness of the potential for depression in this population.

## 5. Conclusion

This study explored family relationships in terms of living arrangements and support networks and also their associations with depression in elderly Korean immigrants. Korean elders who were living independently reported higher levels of depression regardless of the fact that they expressed a desire to live independently and be less dependent upon their adult children. The values of independence and self-reliance widely embraced by the dominant United States culture appeared to be accepted by Korean elders and may be a cause of major life changes. Therefore, a desire for separate households and less dependence on their adult children could be seen as mechanisms of survival in the USA that Korean elders have learned. However, these finding suggest that family support and close relationships with family members still play a very important role in dealing with stressors and in preventing and/or lessening depression.

## Figures and Tables

**Table 1:** Family relationships: description of the sample.

Variables (*N* = 160)	Number (*N*)	Percent (%)
Living Arrangements		

Living with adult children	45	28.2%
Living independently	114	71.3%
Other	1	0.6%

Housing		

Senior Apartment	87	54.4%
House or Apartment belonging to oneself	40	25.0%
House or Apartment belonging to Children	21	19.4%
Other	2	1.3%

Satisfaction with current living arrangement		

Yes	148	92.5%
No	12	7.5%

Living arrangement would be different if in Korea		

Yes	70	43.8%
No	90	56.3%

Preferred living arrangement		

With adult children	36	22.5%
Separate	124	77.5%

Reasons to live in separate household (*N* = 124, 77.5%)		

To protect my privacy and freedom	40	25%
To be less burden on my adult children	20	12.5%
Both	36	22.5%
Others	28	18%
(i) To avoid conflicts with children		
(ii) To maintain ethnic identity, values, and authority		
(iii) To be self-supporting and independent		

Reasons to live with adult children (*N* = 36, 22.5%)		

To be with grandchildren	9	5.6%
Adult children need my help	6	3.8%
Loneliness	5	3.1%
Filial responsibility	4	2.5%
Need support of adult children	4	2.5%
Other	8	5.0%

**Table 2:** Frequent source of social support among elderly Korean immigrants (*N* = 160).

Network member named	Frequency*	Percent (%)
Adult children	507	37.8%
Spouse	203	15.1%
Friends/Neighbors	137	10.2%
Spiritual advisor	41	3.06%
Relatives	19	1.4%
Professional (nurse, doctor, counselor)/Agencies	97	7.24%
No one (prefer to handle it alone)	275	20.5%
No one (no one available)	60	4.48%

Total	1339	100%

*Participants listed more than one source of support.

**Table 3:** Depression among elderly Korean immigrants (*N* = 160).

CES-D Score	Indication	*N*	%
Lower than 16	Not depressed	124	77.5%
16 to 23	Mildly depressed	18	11.3%
24 and higher	Clinically depressed	18	11.3%

## References

[1] Ahn J (2006). Risk factors for depressive symptoms among Korean elderly immigrants. http://sswr.confex.com/sswr/2006/techprogram/P4229.HTM.

[2] Lee MS, Crittenden KS, Yu E (1996). Social support and depression among elderly Korean immigrants in the United States. *International Journal of Aging and Human Development*.

[3] Yamamoto J, Rhee S, Chang DS (1994). Psychiatric disorders among elderly Koreans in the United States. *Community Mental Health Journal*.

[4] Mui A (2001). Stress, coping, and depression among elderly Korean immigrants. *Journal of Human Behavior in the Social Environment*.

[5] Pang KY (1998). Causes of dysphoric experiences among elderly Korean immigrants. *Clinical Gerontologist*.

[6] Ishii-Kuntz M (1997). Intergenerational relationships among Chinese, Japanese, and Korean Americans. *Family Relations*.

[7] Kamo Y, Zhou M (1994). Living arrangements of elderly Chinese and Japanese in the United States. *Journal of Marriage and Family*.

[8] Koh JY, Bell WG (1988). Korean elders in the United States: intergenerational relations and living arrangements. *The Gerontologist*.

[9] Lam RE, Pacala JT, Smith SL (1997). Factors related to depressive symptoms in an elderly Chinese American sample. *Clinical Gerontologist*.

[10] Mackinnon M, Gien L, Durst D (1996). Chinese elders speak out. *Clinical Nursing Research*.

[11] Lee YM (2007). Immigration experience among elderly Korean immigrants. *Journal of Psychiatric and Mental Health Nursing*.

[12] Sin MK, Choe MA, Kim J, Jeon MY (2010). Depressive symptoms in community-dwelling elderly Korean immigrants and elderly Koreans. *Research Gerontological Nursing*.

[13] Lee MS (1989). *Transition, social support, and mental health among older Korean immigrants*.

[14] Lazarus R, Folkman S (1984). *Stress, Appraisal, and Coping*.

[15] Berry JW (1997). Immigration, acculturation and adaptation. *Applied Psychology*.

[16] Folkman S (1984). Personal control and stress an coping processes: a theoretical analysis. *Journal of Personality and Social Psychology*.

[17] Johnson JE (1998). Stress, social support and health in frontier elders. *Journal of Gerontological Nursing*.

[18] Noh S, Avison WR (1996). Immigrants and the stress process: a study of Koreans in Canada. *Journal of Health and Social Behavior*.

[19] Weinert C, Brandt PA (1987). Measuring social support with the personal resources questionnaire. *Nursing Research*.

[20] Radloff LS (1977). The CES-D scale: a self report depression scale for research in the general population. *Applied Psychological Measurement*.

[21] Cho MJ, Kim KH (1998). Use of the center for epidemiologic studies depression (CES-D) scale in Korea. *Journal of Nervous and Mental Disease*.

[22] Shin K (1992). *Correlates of depressive symptomatology in Korean-American Women in New York Cit*.

[23] Administration on Aging (2009). Profile of older Americans: 2009. http://www.aoa.gov/aoaroot/aging_statistics/profile/2009/docs/2009profile_508.pdf.

[24] Gupta R, Yick A (2001). Validation of CES-D scale for older Chinese immigrants. *Journal of Mental Health and Aging*.

[25] Callahan CM, Wolinsky FD, Stump TE, Nienaber NA, Hui SL, Tierney WM (1998). Mortality, symptoms and functional impairment in late-life depression. *Journal of General Internal Medicine*.

[26] Nicholas J, Wiseman M (2009). Elderly poverty and Supplemental Security income. *Social Security Bulletin*.

[27] Kuo WH (1984). Prevalence of depression among Asian-Americans. *Journal of Nervous and Mental Disease*.

